# GDF-15 predicts cardiovascular events in acute chest pain patients

**DOI:** 10.1371/journal.pone.0182314

**Published:** 2017-08-03

**Authors:** Stergios Tzikas, Lars Palapies, Constantinos Bakogiannis, Tanja Zeller, Christoph Sinning, Stephan Baldus, Christoph Bickel, Vassilios Vassilikos, Karl J. Lackner, Andreas Zeiher, Thomas Münzel, Stefan Blankenberg, Till Keller

**Affiliations:** 1 3rd Department of Cardiology, Ippokrateio Hospital, Aristotle University of Thessaloniki, Thessaloniki, Greece; 2 Department of Internal Medicine II, University Medical Center, Johannes Gutenberg University, Mainz, Germany; 3 Division of Cardiology, Department of Medicine III, Goethe University Frankfurt, Frankfurt, Germany; 4 Clinic for General and Interventional Cardiology, University Heart Center Hamburg, Hamburg, Germany; 5 German Center for Cardiovascular Research (DZHK), Berlin, Germany; 6 Department of Internal Medicine III, University of Cologne, Cologne, Germany; 7 Department of Internal Medicine, Federal Armed Forces Hospital, Koblenz, Germany; 8 Institute for Clinical Chemistry and Laboratory Medicine, University Medical Center Mainz, Mainz, Germany; Universitatsklinikum Freiburg, GERMANY

## Abstract

**Background:**

Treatment of patients presenting with possible acute myocardial infarction (AMI) is based on timely diagnosis and proper risk stratification aided by biomarkers. We aimed at evaluating the predictive value of GDF-15 in patients presenting with symptoms suggestive of AMI.

**Methods:**

Consecutive patients presenting with suspected AMI were enrolled in three study centers. Cardiovascular events were assessed during a follow-up period of 6 months with a combined endpoint of death or MI.

**Results:**

From the 1818 enrolled patients (m/f = 1208/610), 413 (22.7%) had an acute MI and 63 patients reached the combined endpoint. Patients with MI and patients with adverse outcome had higher GDF-15 levels compared with non-MI patients (967.1pg/mL vs. 692.2 pg/L, p<0.001) and with event-free patients (1660 pg/mL vs. 756.6 pg/L, p<0.001). GDF-15 levels were lower in patients with SYNTAX score ≤ 22 (797.3 pg/mL vs. 947.2 pg/L, p = 0.036). Increased GDF-15 levels on admission were associated with a hazard ratio of 2.1 for death or MI (95%CI: 1.67–2.65, p<0.001) in a model adjusted for age and sex and of 1.57 (1.13–2.19, p = 0.008) adjusted for the GRACE score variables. GDF-15 showed a relevant reclassification with regards to the GRACE score with an overall net reclassification index (NRI) of 12.5% and an integrated discrimination improvement (IDI) of 14.56% (p = 0.006).

**Conclusion:**

GDF-15 is an independent predictor of future cardiovascular events in patients presenting with suspected MI. GDF-15 levels correlate with the severity of CAD and can identify and risk-stratify patients who need coronary revascularization.

## Introduction

The evaluation of a possible acute myocardial infarction (AMI), including risk stratification of patients presenting with acute chest pain, accounts for numerous patient hospitalizations and remains a major clinical challenge.[1] Biomarkers play a key role in assisting the establishment of the diagnosis, as well as in predicting future cardiovascular risk.[2] An increasing number of emerging biomarkers have been identified to play an important role in the pathophysiology and natural course of acute coronary syndrome (ACS). As a consequence it is becoming crucial to validate their specific predictive value in the particular real-world setting of acute chest pain before implementing them as diagnostic or prognostic tools.[3]

After verifying the right diagnosis, of great importance is the precise and on-time decision for the optimal therapeutic strategy. It has been demonstrated that patients with ST-elevation myocardial infarction (STEMI) will benefit if the reperfusion therapy takes place within the first 2–3 hours of symptom onset.[4]

On the other hand data are not clear for patients with Non-ST-elevation myocardial infarction (NSTEMI). Current guidelines highlight the importance if risk stratification for these patients, which guides the decision invasive versus conservative therapeutic management of the patient. [4]

Growth differentiation factor-15 (GDF-15) is a member of the transforming growth factor β family.[5] Under physiological conditions, it is weakly expressed by endothelial cells and macrophages in most tissues and strongly expressed after tissue injury, inflammation mechanical and oxidative stress.[6–8] In animal models it appears to protect against cardiac injury, possibly because of anti-inflammatory, anti-apoptotic, or antihypertrophic effects.[9–11] In human heart tissue, GDF-15 increases within hours after a myocardial infarction and remains elevated in the infarcted myocardium for several days.[9] Clinical studies in community based populations[12–14] and in patients with manifested cardiovascular disease (CVD)[8, 15–21] have mainly shown higher GDF-15 concentrations to be associated with adverse outcome and disease progression. However whether GDF-15 is a causative mediator or a risk biomarker of CVD remains uncertain. Our primary goal was to test the hypothesis that GDF-15 could predict future cardiovascular events and test its predictive value in comparison to established predictors of cardiovascular risk including the B-type natriuretic peptide, sensitive troponin determination as well as the established Global Registry of Acute Coronary Events (GRACE) risk score.[22] Furthermore, we tried to evaluate the potential relation between GDF-15 levels and the complexity of an underlying CAD quantified by the Synergy Between Percutaneous Coronary Intervention With TAXUS and Cardiac Surgery (SYNTAX) score as well as if GDF-15 could have an additive role in supporting the clinical decision process for the optimal therapeutic strategy (invasive versus conservative).

## Materials and methods

Patients consecutively presenting with symptoms suggestive of an AMI, such as acute chest pain were enrolled in this prospective study as described previously.[23] Patients were enrolled at one of the chest pain units of Johannes Gutenberg-University Medical Center Mainz, the Federal Armed Forces Hospital Koblenz, and the University Hospital Hamburg-Eppendorf. Eligible patients had to be between 18 and 85 years of age, exclusion criteria included major surgery or trauma within the last 4 weeks, pregnancy, intravenous drug abuse and anaemia with haemoglobin level below 10g/dl. The study complies with the Declaration of Helsinki; the local ethics committees of Rheinland-Pfalz and Hamburg approved the study; participation was voluntary and each patient gave written, informed consent.

Venous blood was drawn at admission and after centrifugation, ethylenediaminetetraacetic acid plasma and serum was stored at -80°C until further measurements of the investigational biomarkers. A 12-lead ECG was written at the same time points. Among other data, the classical cardiovascular risk factors were documented at admission as described earlier in detail.[24]

AMI was diagnosed according to the universal definition of MI.[25] Two independent cardiologists defined the final gold-standard diagnosis on the basis of all available clinical, laboratory, and imaging findings. This information includes conventional serial troponin measurements; a detailed description of the used conventional in-house troponin assays is given supplementary (S1 File). In patients with excluded AMI, unstable angina pectoris (UAP) was diagnosed if ECG was non-diagnostic; in-house troponin was negative, but coronary angiography revealed a culprit lesion; or ischemia was proven in stress test with subsequent need of coronary intervention. Patients in whom an AMI or UAP could be excluded were categorized as having non-coronary chest pain (NCCP).

Cardiovascular events were registered during a follow-up period of 6 months. Patients were personally contacted by telephone and/or letter and events were adjudicated through hospital charts, available outpatient information and data from the local civil registry office. The combined endpoint included first occurrence of non-fatal myocardial infarction and/or death.

Routine laboratory parameters including creatinine and C-reactive protein (CRP) were measured immediately after blood withdrawal by standardized methods. The estimated glomerular filtration rate (eGFR) was calculated by the abbreviated MDRD equation.[26]

Growth-differentiation factor 15 (GDF-15) was measured in Serum plasma using a research prototype GDF-15 assay (Abbott Diagnostics, Abbott Park, USA). Limit of detection of the assay is 10pg/mL, intra CV is 5.2%, inter CV is 4.11% and the 99th percentile is 1424.0pg/mL (personal communication Abbott Diagnostics).

Cardiac Troponin I (TnI) was measured using a contemporary sensitive assay on the ADVIA Centaur XP system (TnI-Ultra, Siemens Healthcare Diagnostics, Germany). Assay range is 0.006–50 ng/mL; the coefficient of variation (CV) at 0.03 ng/mL is 10%. The reference limit based on the 99th percentile for a healthy population is 0.04 ng/mL.[27]

B-type natriuretic peptide (BNP) was assayed on the ARCHITECT i System (BNP, Abbott Diagnostics, Germany). The analytical sensitivity of the assay is ≤10 pg/ml with assay range of 0–5000 pg/mL.

Skewed variables were described by median and interquartile range, symmetric variables by mean and standard deviation (SD). The Mann-Whitney test was used to compare biomarker levels among different patient groups. The Spearman correlation coefficient was used to study the association between continuous variables. The relation of the investigational biomarkers to the predefined endpoint was tested with cox regression analyses. Two models were used; a model adjusted for age and gender (M2) and a further model (M3) adjusted for the GRACE score variables: heart rate, (log) creatinine, ST changes in ECG, age, systolic blood pressure and Killip class. Hazard ratios (HR) are given for an increase in one standard deviation of the tested log transformed biomarker. The predictive value of the investigational biomarkers for future cardiovascular events was also assessed with C-indices of both unadjusted models and after adjustment for the above quoted variables. Optimized cut-offs have been calculated as to attain the biggest Youden index (sum of sensitivity and specificity among event and non-event patients minus 1). Survival curves according to different biomarker concentrations were estimated by Kaplan-Meier analysis and compared for significance with the log-rank test. Reclassification with respect to GRACE model was assessed with categorical and continuous net reclassification improvement (NRI), integrated Discrimination Improvement (IDI) and relative IDI. P<0.05 was considered statistically significant. Analyses were carried out using R 3.1.1 (R Foundation for Statistical Computing, Vienna, Austria).

## Results

**Fig 1:**
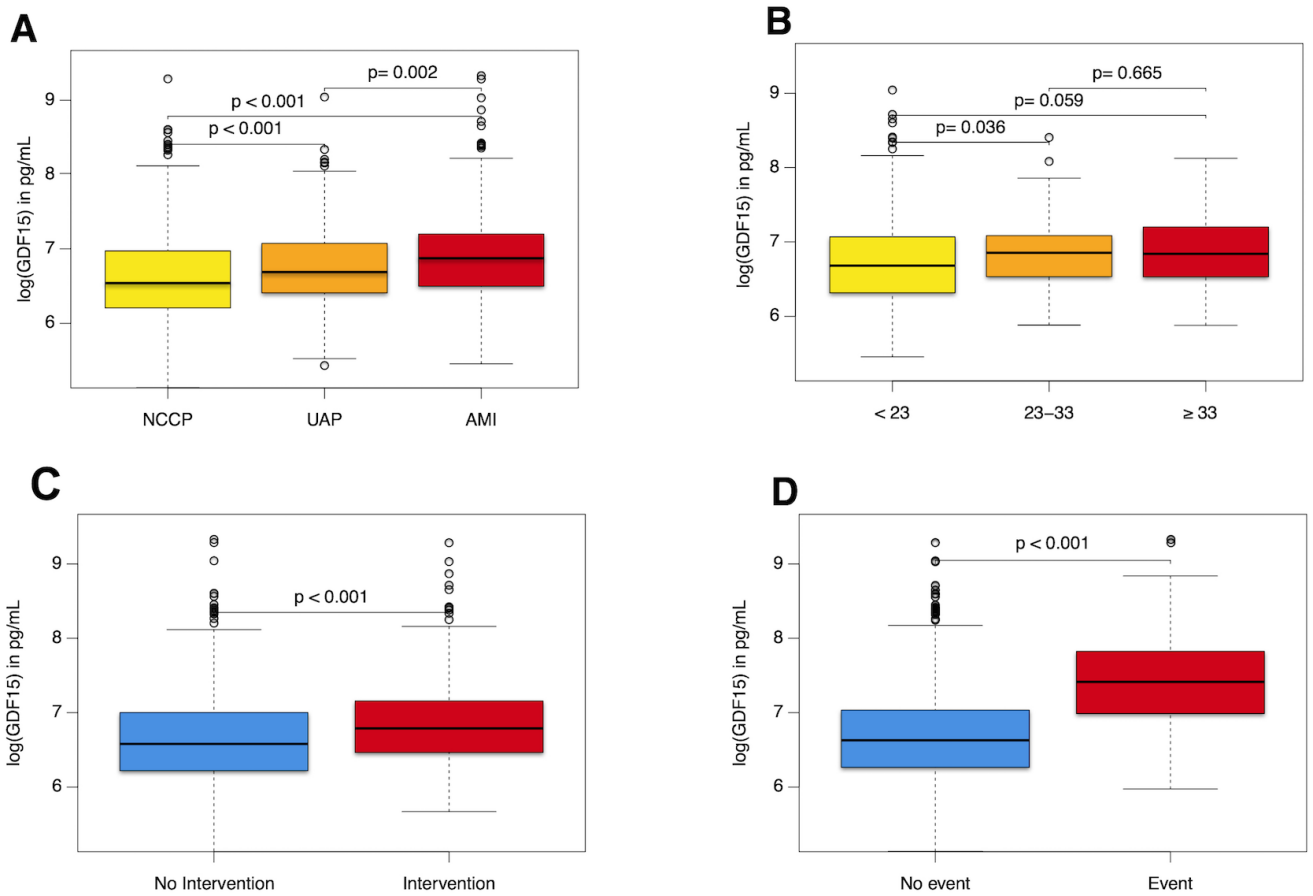
Box plots of GDF-15. Presented are box plots of GDF-15 stratified for A) diagnosis B) SYNTAX Score C) need for Intervention and D) occurrence of the endpoint.

From the 1818 enrolled patients (m/f = 1208/610) with suspected AMI, 413 (22.7%) had an AMI (22.7%), with 130 patients (7.2%) having a STEMI and 283 patients (15.6%) NSTEMI. Unstable angina pectoris was diagnosed in 240 (13.2%) patients (see also S1 Table). Biomarkers of myocardial necrosis as well as GDF-15 levels (967.10 pg/mL vs. 692.15 pg/mL, p<0.001) were significantly higher in patients suffering an AMI compared to NCCP patients (see also Fig 1A). Six-month follow-up information was available in 1804 (99.2%) patients with total follow-up time of 200 days. 63 individuals reached the combined endpoint of Death and/or MI (32 deaths and 31 non-fatal MIs). Individuals that reached the combined endpoint had significantly higher GDF-15 levels at baseline (1660 pg/mL vs. 756 pg/mL, p<0.001) than those with event-free survival. Baseline characteristics of the study sample stratified by the final outcome are summarized as Table 1.

**Table 1:** Baseline characteristics of the study cohort according to outcome.

	All	No Event	Event	p-value
No. of patients (%)	1804 (100)	1741 (96.5)	63 (3.5%)	
Age (years)	62 ± 13	61 ± 13	72 ± 10	< 0.001
Female gender (%)	607 / 1804 (34)	591 / 1741 (34)	16 / 63 (25)	0.202
**Risk factors**				
Hypertension (%)	1330 / 1804 (74)	1273 / 1741 (73)	57 / 63 (90)	0.003
Dyslipidemia (%)	1320 / 1804 (73)	1278 / 1741 (73)	42 / 63 (67)	0.298
Diabetes mellitus (%)	341 / 1802 (19)	314 / 1739 (18)	27 / 63 (43)	< 0.001
Obesity (BMI>30) (%)	464 / 1681 (28)	449 / 1619 (28)	15 / 62 (24)	0.64
Active Smoker (%)	446 / 1800 (25)	438 / 1737 (25)	8 / 63 (13)	0.035
Former Smoker (%)	579 / 1729 (33)	548 / 1666 (33)	31 / 63 (49)	0.011
**History**				
Family History of CAD (%)	626 / 1800 (35)	612 / 1737 (35)	14 / 63 (22)	0.046
History of MI (%)	429 / 1799 (24)	400 / 1736 (23)	29 / 63 (46)	< 0.001
Known CAD (%)	671 / 1802 (37)	633 / 1739 (36)	38 / 63 (60)	< 0.001
**Laboratory parameters**				
Total cholesterol (mg/dL)	196 (164.5/228)	196 (165/228.5)	188.5 (144.5/225.5)	0.156
LDL cholesterol (mg/dL)	116 (90/146)	117 (90/146)	106 (79.5/154)	0.574
HDL cholesterol (mg/dL)	48 (40/59)	48 (40/59)	44 (37/52.8)	0.057
Troponin I (pg/mL)	0.012 (0.004/0.066)	0.012 (0.003/0.058)	0.196 (0.047/3.003)	< 0.001
BNP (pg/mL)	33.8 (12.2/105.4)	32.9 (11.7/98.1)	164.1 (54/439)	< 0.001
CRP (mg/L)	2.5 (1.3/5.8)	2.5 (1.2/5.5)	5.6 (2.3/13)	< 0.001
eGFR (mL/min for 1.73m^2^)	79.4 (66.5/91.5)	79.8 (67.4/92)	60.6 (43.1/74.1)	< 0.001
GDF-15 (pg/mL)	774.1 (535/1163)	756.6 (525.8/1133)	1660 (1084/2502)	< 0.001

Data presented as number (percentage) of patients, mean ± standard deviation for even variables, or median and 25th/75th interquartile range for skewed variables. BMI denotes body mass index, MI denotes myocardial infarction, eGFR denotes estimated glomerular filtration rate, CAD denotes coronary artery disease, LDL denotes low-density lipoprotein, HDL denotes high-density lipoprotein, CRP denotes C-reactive protein, BNP denotes B-type natriuretic peptide, GDF denotes growth differentiation factor.

At baseline, median GDF-15 levels were not influenced by gender (males vs. females with 776.3 vs. 764.1 pg/mL, p>0.5) but were significantly higher in patients with known arterial hypertension (854.3 vs. 571.6 pg/mL, p<0.001) and in diabetics (1142 vs. 704.9 pg/mL, p<0.001). Furthermore, non-smokers showed higher GDF-15 values (785.6 vs. 730.9 pg/mL of smokers, p = 0.01).

**Fig 2:**
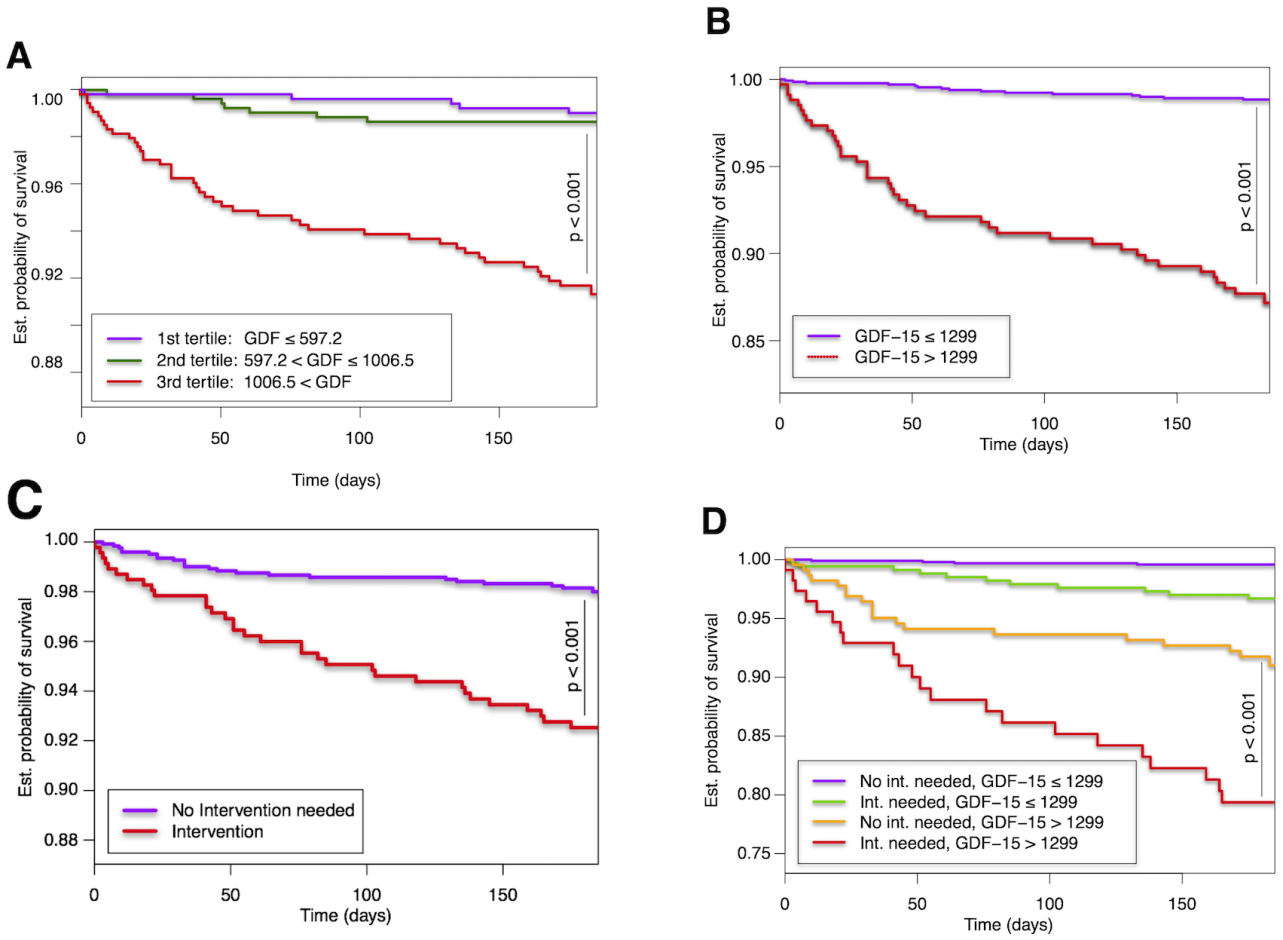
Cumulative survival curves according to various strata. Presented are Kaplan-Meier survival curves in patients presenting with chest pain for the combined primary endpoint of death and non-fatal myocardial infarction within 6 months after presentation according to A) tertiles of GDF-15 levels and B) levels dichotomized using an optimized cut-off of 1298.9 pg/mL and C) need for intervention D) the optimized cut-off and need for intervention.

Kaplan Meier analyses show a relevant association of baseline GDF-15 values with 6-month outcome if using GDF-15 tertiles (Fig 2A) or dichotomized using an optimized cut-off of 1298.9 pg/mL (Fig 2B). The prognostic value of GDF-15 is also supported by Cox regression analysis. GDF-15 adjusted for age and gender was associated with a Hazard ratio (HR) of 2.1 (95%CI: 1.67–2.65, p<0.001) for the risk of death or MI within 6 months after admission and a HR of 1.57 (95%CI: 1.13–2.19, p = 0.008) if adjusted for the GRACE score risk variables. As comparators, BNP was associated with a HR of 1.94 (1.45–2.60, p<0.001) and 1.45 (95%CI: 1.06–1.98, p = 0.021) and Troponin I with a HR of 1.97 (95%CI: 1.62–2.40, p<0.001) and 1.88 (95%CI: 1.52–2.34, p<0.001) respectively (see also S3 Table).

Correspondingly, GDF-15 at admission showed an association with Death or MI in an age and gender adjusted model with a C-index of 0.80 (95%CI: 0.72–0.88). Of note, BNP showed in the same model a C-index of 0.78 (95%CI: 0.71–0.86). C-indices of different models (that contain age and gender as well as the GRACE score variables) including various biomarkers are depicted in S4 Table.

The variables of the established GRACE score, as well as the GRACE score itself in case of the categorical net reclassification improvement (NRI, with a reference cut-off for the GRACE score of 141 points = 21% of risk, as used before[28]), were used to estimate risk of the enrolled patients with suspected ACS. Net reclassification for the addition of baseline GDF-15 amounted to an expected number of 42 events (286 non-events) among patients put at higher risk as well as 31 expected events (296 non-events) among patients put at lower risk than indicated by the GRACE score. The overall respective NRI was 12.5% (p = 0.045) and the relative integrated discrimination improvement (IDI) was 14.56% (p = 0.006). Similar results were observed with BNP with an overall NRI of 10% and a relative IDI of 7.69% (p = 0.028). (See S5 & S6 Tables for complete reclassification data).

The complexity of a potentially underlying CAD was quantified by the SYNTAX score. Baseline characteristics of the study sample stratified by the severity of CAD are summarized as S2 Table. Specifically, GDF-15 values tended to be higher in patients with more complex coronary anatomy (see also Fig 1B). A moderate positive correlation of GDF-15 and BNP (rho = 0.453, p<0.001) and a moderate negative correlation with eGFR (rho = -0.464, p<0.001) were observed. GDF-15 and CRP as well as GDF-15 and Troponin I correlated only weakly together (rho = 0.304 and 0.299 respectively, with p<0.001 for both)(see also Table 2 on correlations stratified by CAD complexity). Additionally, median GDF-15 concentrations according to categorical risk factors stratified by CAD complexity are given as Table 3.

**Table 2:** Correlations of continuous variables with GDF-15 concentrations.

		All	Low Syntax Score: < 23	Intermediate Syntax Score: 23–33	High Syntax Score: ≥ 33
Total cholesterol	Corr.coef.	-0.126	-0.136	-0.27	-0.103
	p-value	< 0.001	< 0.001	0.04	> 0.5
LDL	Corr.coef.	-0.125	-0.108	-0.277	-0.047
	p-value	< 0.001	0.02	0.03	> 0.5
HDL	Corr.coef.	-0.095	-0.112	0.054	0.001
	p-value	< 0.001	0.02	> 0.5	> 0.5
eGFR	Corr.coef.	-0.464	-0.408	-0.612	-0.524
	p-value	< 0.001	< 0.001	< 0.001	0.01
Troponin I	Corr.coef.	0.299	0.176	0.169	-0.162
	p-value	< 0.001	< 0.001	0.18	0.44
BNP	Corr.coef.	0.453	0.323	0.466	0.345
	p-value	< 0.001	< 0.001	< 0.001	0.09
CRP	Corr.coef.	0.304	0.284	0.194	0.017
	p-value	< 0.001	< 0.001	0.12	> 0.5

Presented are the Spearman correlations of GDF-15 with different continuous variables. LDL denotes low-density lipoprotein, HDL denotes high-density lipoprotein, BNP denotes B-type natriuretic peptide, eGFR denotes estimated glomerular filtration rate and CRP denotes C—reactive protein.

**Table 3:** Concentrations of GDF-15 according to categorical risk factors stratified for the SYNTAX Score.

	All	p	Low Syntax Score: < 23	p	Intermediate Syntax Score: 23–33	p	High Syntax Score: ≥ 33	p
Male	776.3	>0.5	804.4	>0.5	906.2	0.09	934.6	>0.5
Female	764.1		794.1		1248.3		956.7	
BMI < 30	756.6	0.03	767	0.02	967.1	0.14	971.8	>0.5
BMI > 30	806.6		890.4		842.3		892.9	
No Hypertension	571.6	<0.001	691.3	0.001	957.2	>0.5	814.4	>0.5
Hypertension	854.3		847.1		920.6		971.8	
No Diabetes	704.9	<0.001	740.5	<0.001	903	0.03	892.9	0.1
Diabetes	1142		1242		1142.1		1341	
No smoking	785.6	0.01	794.1	>0.5	1017.5	0.02	892.9	0.19
Smoking	730.9		807.7		698.1		1143	
No Dyslipidemia	712.2	0.002	782.8	>0.5	987.1	>0.5	1431.7	0.07
Dyslipidemia	797.8		800		920.6		892.9	
No CAD	673.7	<0.001	753.9	0.003	947.2	>0.5	904.4	0.47
Known CAD	947.7		893.1		941.4		1281.1	

Presented are the medians for each group, which were tested for equality by the Mann-Whitney test. CAD denotes coronary artery disease. BMI denotes body mass index.

As “real life” cohort, the decision if a patient will undergo a coronary intervention or not was based on clinical and laboratory data and upon the physician’s discretion. Of the 1818 patient enrolled, 502 patients underwent a percutaneous coronary intervention (PCI) due to the symptom leading to presentation. The majority of these patients (71%) were initially diagnosed with an acute myocardial infarction and had a poorer outcome compared to patients not in need for a PCI, likewise to patients with increased GDF-15 levels. Interestingly, patients who underwent a coronary intervention had significantly higher GDF-15 levels compared to patients who had no intervention. Kaplan-Meier analysis revealed that GDF-15 had a strong prognostic role demonstrating that patients who underwent an intervention and had higher GDF-15 levels were at significantly higher risk, compared to other patient groups. (Fig 2C)(compare Fig 1D).

Patients requiring a coronary intervention had higher baseline GDF-15 levels with 887.1 pg/mL vs. 721.4 pg/mL (p<0.001) (Fig 1C). Furthermore, by using the optimized cut-off of 1298.9 pg/ml, we could divide study population in two groups based on GDF-15 baseline values. Patients who had higher GDF-15, above the cut-off value, and were in need of PCI had significantly higher mortality risk, compared to other patient groups. (Fig 2D).

## Discussion

Biomarkers changed our diagnostic and therapeutic approach of ACS. New generation highly sensitive troponins facilitate an early and accurate diagnosis of ACS. On the other hand, because of the higher sensitivity, more patients are gradually rated as higher risk compared to the past, resulting in a more aggressive therapeutic approach, generating a lot of controversies. [29–31] Emerging biomarkers could also be helpful especially in risk stratification of these patients, supporting the optimization of the treatment plan. GDF-15 was originally cloned on the basis of its enhanced expression during macrophage activation and has been associated to inflammatory states in many tissues.[32] Various key studies have already reported on GDF-15 in the settings of cardiovascular disease. Kempf et al. reported that GDF-15 provides prognostic information beyond established clinical and biochemical markers in STEMI patients.[16] This study included patients presenting with STEMI recruited in the late 90’s for a thrombolysis study (tenecteplase with front-loaded alteplase) and reported a strong association of elevated GDF-15 levels with 1 year mortality. Kempf et al. also reported on the prognostic value of GDF-15 in the settings of chronic heart failure supporting the predictive value of this biomarker.[17]

In our study we included patients admitted with suspected ACS. Our primary goal was to investigate the potent prognostic role of GDF-15 levels in a “real-world” population. For this reason we conducted a six-months follow-up of our patients, with total follow-up time of 200 days. Our results demonstrated that baseline GDF-15 levels were significantly higher at patients with AMI compared to UAP and patients with NCCP. Furthermore patients who reached the combined endpoint (death or MI), during the six-months follow-up time, had significantly higher GDF-15 levels. In addition higher baseline GDF-15 levels were positively correlated with higher mortality risk. In the same year, Schaub et al. reported on the prognostic value of GDF-15 on data collected from a smaller cohort (n = 646), but very similar to the one we used.[33] That study was the first to be conducted on an unselected chest pain population, although the investigators excluded patients with terminal renal disease from the investigation. Our data confirm the results of Schaub *et al*. in a larger unselected cohort and expand these results regarding CAD complexity and choice of interventional strategy.

In addition we evaluated the possible association of GDF-15 with the complexity of the coronary disease. Our data revealed that patients with higher SYNTAX score had also higher GDF-15 levels. Moreover, as it was expected, patients who needed an intervention, had also higher GDF-15 levels. Patients who underwent an intervention and had higher GDF-15 levels were at significantly higher risk compared to patients who had no intervention, or had an intervention but low GDF-15 levels. Wollert et al. described first a graded relationship between the levels of GDF-15 and the effects of the invasive strategy on the composite and individual end points[8] supporting our data. This study used samples and outcome data from the *Fragmin and Fast Revascularization during Instability in Coronary Artery Disease* (FRISC-II) study conducted in the late ‘90s, used a third generation Troponin assay and showed a significant interaction between GDF-15 levels and the effect of treatment strategy on the recurrent MI. Interestingly the predefined cut-off used in that study (1200 ng/L) was pretty closed to the optimized one (1298.9 ng/L) calculated in our population.

Also in line with our data, here on the prognostic value of GDF-15, Widera et al. showed that a single GDF-15 measurement on admission markedly enhances the predictive value of the well-known GRACE risk score in NSTE-ACS patients, performing even better as NT-proBNP.[34] Our results not only support these findings but also extend them also to the evaluation of BNP, which is also outperformed by GDF-15 when added to the GRACE risk score. This might indeed have clinical implications, as the latter is a recognized risk stratification tool for the choice of invasive treatment.[28]

Recently, another study by Wallentin et al. investigated the prognostic relevance of GDF-15 in relation to randomized anti-platelet treatment and an interventional management strategy in the NSTE-ACS subgroup of the *Platelet Inhibition and Patient Outcomes* (PLATO) trial.[35] In that study, GDF-15 was a significant predictor of cardiovascular death, myocardial infarction, and stroke in patients managed both conservative and invasively. Interestingly, the magnitude of benefit of ticagrelor vs. clopidogrel was related to the degree of elevation of GDF-15, pointing towards GDF-15 as a useful biomarker for treatment decisions. Furthermore, in the same population, it has been recently demonstrated that GDF-15 has a significant prognostic value in patients with NSTE-ACS undergoing revascularization. GDF-15 levels were directly associated with the extent of CAD in these patients and GDF-15 when added to the prediction models led to reclassification of patients at lower risk level, while NT-proBNP could better identify NSTE-ACS patients at higher risk level. [36]

The present study was performed in a representative German sample; Distribution may differ for other regions. A relatively small number of events have been registered during follow-up in this intermediate risk cohort, which might lead to unstable results especially in the multivariable survival analysis. The clinical benefit from improved risk stratification was not assessed from the present study and can only be hypothesized. The calculation of p-values for the comparison of risk groups defined by biomarker levels is potentially impaired by the fact that corresponding cut-offs have been chosen in retrospect against test assumptions.

GDF-15 is a significant predictor of future cardiovascular events in patients presenting with acute chest pain and might be a useful biomarker for risk stratification of patients presenting with signs and symptoms of ACS in an era where there is still an unmet need for better prognostication models that could identify high-risk patients and thus better guide our treatment decisions.

Whether novel biomarkers or combination of biomarkers can address the treatment dilemma of an invasive versus conservative approach, particularly in this subgroup of patients who are reclassified from unstable angina to NSTE myocardial infarction remains to be seen. Potential benefits of using GDF-15 in risk stratification and triaging of chest pain patients have to be further explored in future randomized interventional studies.

## Supporting information

S1 File Supplementary methods. (DOC)

S1 Table Baseline characteristics of the study sample stratified by diagnosis. Data presented as number (percentage) of patients, mean ± standard deviation for even variables, or median and 25th/75th interquartile range for skewed variables. NCCP denotes non-coronary chest pain, UAP denotes unstable angina pectoris, AMI denotes acute myocardial infarction, eGFR denotes estimated glomerular filtration rate. CK denotes creatine kinase, CKMB denotes creatine kinase MB. CAD denotes coronary artery disease, LDL denotes low-density lipoprotein, HDL denotes high-density lipoprotein, CRP denotes C-reactive protein, BNP denotes B-type natriuretic peptide, GDF denotes growth differentiation factor.
(DOC)

S2 Table Baseline characteristics of the study cohort according to severity of CAD. Data presented as number (percentage) of patients, mean ± standard deviation for even variables, or median and 25th/75th interquartile range for skewed variables. BMI denotes body mass index, MI denotes myocardial infarction, eGFR denotes estimated glomerular filtration rate, CAD denotes coronary artery disease, LDL denotes low-density lipoprotein, HDL denotes high-density lipoprotein, CRP denotes C—reactive protein, BNP denotes B-type natriuretic peptide, GDF denotes growth differentiation factor.
(DOC)

S3 Table Cox Regression Analysis of biomarkers measured at admission. Presented are hazard ratios per standard deviation increase using the biomarker as continuous value (A) and (B) HR based on calculated optimized thresholds (written as binary quantities indicating whether value is above Youden-optimized threshold). Event was defined as Death and/or MI in the six months follow up. There were 63 events. All biomarkers entered the regressions after being log-transformed (except eGFR). [M2] was age and sex adjusted. [M3] was adjusted for the GRACE score variables: heart rate, (log) creatinine, ST changes in ECG, age, systolic blood pressure and Killip class.
(DOC)

S4 Table C-indices of biomarkers measured at admission. C-indices are given, together with 95% confidence intervals. Event was defined as Death+MI in six months FU. All biomarkers entered the regressions after being log-transformed (except eGFR). [M2] was age and sex adjusted with C-index of basemodel of 0.76 (0.69, 0.83). [M3] was adjusted for the GRACE score variables: heart rate, (log) creatinine, ST changes in ECG, age, systolic blood pressure and Killip class with C-index of basemodel of 0.83 (0.75, 0.90). The p-value of C-index comparing TnI and GDF15 in M1 is >0.5, in M2 is >0.5 and in M3 is >0.5.
(DOC)

S5 Table Reclassification with regards to GRACE model. NRI denotes net reclassification index. eGFR denotes estimated glomerular filtration rate, BNP denotes B-type natriuretic peptide, GDF denotes growth differentiation factor.
(DOC)

S6 Table Reclassification for adding biomarkers to GRACE score. NRI denotes net reclassification index. Event was defined as Death or myocardial infarction in the six months following baseline presentation.
(DOC)
