# Supplementary material for: GDF-15 predicts cardiovascular events in acute chest pain patients
Source: PLoS One. 2017 Aug 3;12(8):e0182314. doi: 10.1371/journal.pone.0182314 (PMC5542604; doi:10.1371/journal.pone.0182314)
Supplement: S1 File — (DOC) [file pone.0182314.s001.doc]

### **S1 File.**

### Conventional troponin assays used for the adjudication of the final diagnosis based on all serial troponin measurements were: cardiac troponin T in Mainz and Hamburg and cardiac troponin I in Koblenz. In-house troponin was measured immediately after blood withdrawal on admission. In-house troponin was represented at two study centers by cardiac troponin T measurement (4th generation Elecsys 2010 TnT assay, Roche Diagnostics, Germany). Detection limit of the assay is 0.01ng/mL with measuring range of 0.01-25ng/mL. Reference limit based on the 99th percentile for a healthy population is 0.01ng/mL and 10% coefficient of variation (CV) is represented by 0.03ng/mL used as diagnostic cut-off. At one study center, in-house troponin is represented by cardiac troponin I measurement (Dimension RxL TnI, Siemens Healthcare Diagnostics, Germany). This assay has a detection limit of 0.04ng/mL with measuring range of 0.04-40ng/mL. The 99th percentile is 0.07ng/mL and the 10% CV used as diagnostic cut-off is 0.14ng/mL.
