# Supplementary material for: GDF-15 predicts cardiovascular events in acute chest pain patients
Source: PLoS One. 2017 Aug 3;12(8):e0182314. doi: 10.1371/journal.pone.0182314 (PMC5542604; doi:10.1371/journal.pone.0182314)
Supplement: S1 Table — Data presented as number (percentage) of patients, mean ± standard deviation for even variables, or median and 25th/75th interquartile range for skewed variables. NCCP denotes non-coronary chest pain, UAP denotes unstable angina pectoris, AMI denotes acute myocardial infarction, eGFR denotes estimated glomerular filtration rate. CK denotes creatine kinase, CKMB denotes creatine kinase MB. CAD denotes coronary artery disease, LDL denotes low-density lipoprotein, HDL denotes high-density lipoprotein, CRP denotes C-reactive protein, BNP denotes B-type natriuretic peptide, GDF denotes growth differentiation factor. (DOC) [file pone.0182314.s002.doc]

### **S1 Table.**

|  | **NCCP** | **UAP** | **AMI** | **All** |
| --- | --- | --- | --- | --- |
| No. of patients (%) | 1165 (64.1) | 240 (13.2) | 413 (22.7) | 1818 (100) |
| Age (years) | 59.7 ± 14.3 | 65.2 ± 10.5 | 64.0 ± 11.8 | 61.4 ± 13.5 |
| Male gender (%) | 729 (62.6) | 165 (68.8) | 314 (76.0) | 1208 (66.4) |
| **Risk factors** |  |  |  |  |
| Body mass index (kg/m²) | 27.7 ± 4.9 | 27.9 ± 4.4 | 27.9 ± 4.6 | 27.8 ± 4.8 |
| Hypertension (%) | 822 (70.6) | 204 (85.0) | 313 (75.8) | 1339 (73.7) |
| Diabetes mellitus (%) | 140 (12.5) | 53 (23.0) | 80 (20.0) | 273 (15.6) |
| Smoking status |  |  |  |  |
| Current smoker (%) | 254 (21.9) | 40 (17.1) | 143 (35.1) | 437 (24.3) |
| Former smoker (%) | 326 (28.5) | 76 (33.2) | 124 (31.0) | 526 (29.7) |
| Never smoker (%) | 564 (49.3) | 111 (48.9) | 133 (33.2) | 808 (45.6) |
| Dyslipidaemia (%) | 824 (70.7) | 193 (80.4) | 311 (75.3) | 1328 (73.0) |
| **History** |  |  |  |  |
| Family History of CAD (%) | 379 (33.4) | 71 (32.3) | 118 (29.9) | 568 (32.5) |
| Known CAD (%) | 361 (31.8) | 137 (58.5) | 136 (33.7) | 634 (35.8) |
| **Laboratory parameters** |  |  |  |  |
| Total cholesterol (mg/dL) | 197.7 ± 49.0 | 196.7 ± 47.5 | 205.0 ± 50.1 | 199.2 ± 49.1 |
| HDL cholesterol (mg/dL) | 51.8 ± 15.9 | 49.3 ± 14.0 | 47.6 ± 13.8 | 50.5 ± 15.3 |
| LDL cholesterol (mg/dL) | 117.2 ± 40.8 | 116.9 ± 41.9 | 129.6 ± 43.8 | 119.9 ± 41.9 |
| Troponin I (pg/mL) | 4.8 (1.7/9.0) | 9.0 (4.5/26.0) | 271.5 (60.1/1864.5) | 7.4 (3.5/37.8) |
| CK (U/L) | 74.0 (51.0/114.0) | 74.0 (53.0/108.0) | 121.0 (79.0/223.7) | 82.0 (56.0/131.0) |
| CKMB (ng/mL) | 1.00 (0.60/1.50) | 1.10 (0.70/1.70) | 3.30 (1.50/10.50) | 1.200 (0.70/2.00) |
| BNP (pg/mL) | 26.4 (9.1, 68.9) | 47.9 (17.3, 105.9) | 80.3 (23.7, 238.6) | 33.8 (12.1, 105.3) |
| CRP (mg/L) | 2.3 (1.1/5.4) | 2.3 (1.3/4.5) | 3.4 (1.7/8.8) | 2.5 (1.3/5.8) |
| Creatinine | 0.94 (0.82/1.08) | 0.93 (0.82/1.06) | 0.99 (0.88/1.16) | 0.95 (0.83/1.09) |
| eGFR (mL/min for 1.73m²) | 80.1 ± 21.0 | 79.8 ± 21.1 | 75.5 ± 22.3 | 79.0 ± 21.4 |
| GDF15 (pg/mL) | 692.15 (495.76/1068.63) | 803.75 (606.58/1182.11) | 967.10 (662.97/1340.53) | 772.90 (534.80/1164.23) |
| **Time of chest pain onset** |  |  |  |  |
| < 3h (%) | 446 (38.28) | 84 (35.00) | 166 (40.19) | 696 (38.28) |
| < 6h (%) | 693 (59.48) | 139 (57.92) | 237 (57.38) | 1069 (58.80) |
| < 12h (%) | 877 (75.28) | 171 (71.25) | 289 (69.98) | 1337 (73.54) |
| ≥12h (%) | 288 (24.72) | 69 (28.75) | 124 (30.02) | 481 (26.46) |
