# Supplementary material for: GDF-15 predicts cardiovascular events in acute chest pain patients
Source: PLoS One. 2017 Aug 3;12(8):e0182314. doi: 10.1371/journal.pone.0182314 (PMC5542604; doi:10.1371/journal.pone.0182314)
Supplement: S2 Table — Data presented as number (percentage) of patients, mean ± standard deviation for even variables, or median and 25th/75th interquartile range for skewed variables. BMI denotes body mass index, MI denotes myocardial infarction, eGFR denotes estimated glomerular filtration rate, CAD denotes coronary artery disease, LDL denotes low-density lipoprotein, HDL denotes high-density lipoprotein, CRP denotes C—reactive protein, BNP denotes B-type natriuretic peptide, GDF denotes growth differentiation factor. (DOC) [file pone.0182314.s003.doc]

### **S2 Table.**

|  | **All** | **Low Syntax Score: < 23** | **Intermediate Syntax Score: 23-33** | **High Syntax Score: ≥ 33** |
| --- | --- | --- | --- | --- |
| No. of patients (%) | 619 | 526 (85) | 67 (11) | 26 (4) |
| Female gender (%) | 201 / 619 (32) | 187 / 526 (36) | 10 / 67 (15) | 4 / 26 (15) |
| Age (years) | 63 ± 12 | 62 ± 12 | 66 ± 12 | 65 ± 11 |
| **Risk factors** |  |  |  |  |
| Hypertension (%) | 467 / 619 (75) | 392 / 526 (75) | 53 / 67 (79) | 22 / 26 (85) |
| Dyslipidemia (%) | 489 / 619 (79) | 413 / 526 (79) | 53 / 67 (79) | 23 / 26 (88) |
| Diabetes (%) | 110 / 619 (18) | 86 / 526 (16) | 19 / 67 (28) | 5 / 26 (19) |
| Obesity (BMI>30) (%) | 165 / 590 (28) | 134 / 503 (27) | 23 / 64 (36) | 8 / 23 (35) |
| Active Smoker (%) | 183 / 619 (30) | 151 / 526 (29) | 22 / 67 (33) | 10 / 26 (38) |
| Former Smoker (%) | 194 / 589 (33) | 168 / 500 (34) | 20 / 63 (32) | 6 / 26 (23) |
| Family History of CAD (%) | 212 / 618 (34) | 180 / 525 (34) | 24 / 67 (36) | 8 / 26 (31) |
| **History** |  |  |  |  |
| History of MI (%) | 118 / 618 (19) | 97 / 525 (18) | 14 / 67 (21) | 7 / 26 (27) |
| Known CAD (%) | 185 / 619 (30) | 156 / 526 (30) | 23 / 67 (34) | 6 / 26 (23) |
| **Laboratory parameters** |  |  |  |  |
| Total cholesterol (mg/dL) | 204.5 (175/239) | 203 (175/238.8) | 204 (169/231) | 235 (215/251.5) |
| LDL cholesterol (mg/dL) | 126 (99/157) | 125 (99/156) | 133 (93/149) | 153 (127/170) |
| HDL cholesterol (mg/dL) | 48 (39/58) | 48 (40/59.2) | 44 (39/56) | 45 (38.5/55) |
| Troponin I (pg/mL) | 0.061 (0.01/0.705) | 0.048 (0.008/0.49) | 0.108 (0.036/2.141) | 0.908 (0.105/3.518) |
| BNP (pg/mL) | 46.7 (16.4/138.6) | 39.3 (15.5/119) | 81.9 (23.9/169.7) | 168.6 (61.2/358.3) |
| CRP (mg/L) | 2.6 (1.4, 6.6) | 2.6 (1.3, 6.1) | 2.5 (1.8, 6.6) | 5.2 (2.2, 14) |
| eGFR (mL/min for 1.73m²) | 78.2 (66/90.9) | 78.3 (65.7/91.4) | 77.1 (65.2/91.6) | 76.6 (69.3/87.9) |
| GDF-15 (pg/mL) | 830.4 (563/1186) | 797.3 (553.5/1172) | 947.2 (688.5/1195) | 934.6 (685.6/1341) |
