# Supplementary material for: GDF-15 predicts cardiovascular events in acute chest pain patients
Source: PLoS One. 2017 Aug 3;12(8):e0182314. doi: 10.1371/journal.pone.0182314 (PMC5542604; doi:10.1371/journal.pone.0182314)
Supplement: S3 Table — Presented are hazard ratios per standard deviation increase using the biomarker as continuous value (A) and (B) HR based on calculated optimized thresholds (written as binary quantities indicating whether value is above Youden-optimized threshold). Event was defined as Death and/or MI in the six months follow up. There were 63 events. All biomarkers entered the regressions after being log-transformed (except eGFR). [M2] was age and sex adjusted. [M3] was adjusted for the GRACE score variables: heart rate, (log) creatinine, ST changes in ECG, age, systolic blood pressure and Killip class. (DOC) [file pone.0182314.s004.doc]

### **S3 Table.**

| **A** | **HR (95%CI) 1 SD Inc. [M2]** | **p-value [M2]** | **HR (95%CI) 1 SD Inc. [M3]** | **p-value [M3]** |
| --- | --- | --- | --- | --- |
| Troponin I | 1.97 (1.62, 2.4) | < 0.001 | 1.88 (1.52, 2.34) | < 0.001 |
| Creatinine kinase | 1.33 (1.09, 1.64) | 0.006 | 1.27 (1.03, 1.56) | 0.024 |
| Creatine kinase-MB | 1.51 (1.26, 1.8) | < 0.001 | 1.45 (1.2, 1.75) | < 0.001 |
| eGFR | 0.51 (0.4, 0.67) | < 0.001 | 0.8 (0.48, 1.34) | 0.403 |
| GDF15 | 2.1 (1.67, 2.65) | < 0.001 | 1.57 (1.13, 2.19) | 0.008 |
| BNP | 1.94 (1.45, 2.6) | < 0.001 | 1.45 (1.06, 1.98) | 0.021 |

| **B** | **HR (95%CI) [M2]** | **p-value [M2]** | **HR (95%CI) [M3]** | **p-value [M3]** |
| --- | --- | --- | --- | --- |
| Troponin I | 2.48 (1.87, 3.28) | < 0.001 | 2.34 (1.74, 3.15) | < 0.001 |
| Creatinine kinase | 1.52 (1.23, 1.87) | < 0.001 | 1.44 (1.16, 1.79) | < 0.001 |
| Creatine kinase-MB | 1.96 (1.37, 2.81) | < 0.001 | 1.9 (1.32, 2.72) | < 0.001 |
| eGFR | 1.79 (0, Inf) | > 0.5 | 1.91 (0, Inf) | > 0.5 |
| GDF-15 | 2.14 (1.67, 2.74) | < 0.001 | 1.85 (1.4, 2.43) | < 0.001 |
| BNP | 1.69 (1.24, 2.3) | < 0.001 | 1.54 (1.11, 2.13) | 0.01 |
