# Supplementary material for: GDF-15 predicts cardiovascular events in acute chest pain patients
Source: PLoS One. 2017 Aug 3;12(8):e0182314. doi: 10.1371/journal.pone.0182314 (PMC5542604; doi:10.1371/journal.pone.0182314)
Supplement: S4 Table — C-indices are given, together with 95% confidence intervals. Event was defined as Death+MI in six months FU. All biomarkers entered the regressions after being log-transformed (except eGFR). [M2] was age and sex adjusted with C-index of basemodel of 0.76 (0.69, 0.83). [M3] was adjusted for the GRACE score variables: heart rate, (log) creatinine, ST changes in ECG, age, systolic blood pressure and Killip class with C-index of basemodel of 0.83 (0.75, 0.90). The p-value of C-index comparing TnI and GDF15 in M1 is >0.5, in M2 is >0.5 and in M3 is >0.5. (DOC) [file pone.0182314.s005.doc]

### **S4 Table**.

|  | **C-Index [M2]** | **C-Index [M3]** |
| --- | --- | --- |
| Troponin I | 0.82 (0.74, 0.89) | 0.85 (0.78, 0.93) |
| CK | 0.76 (0.69, 0.84) | 0.83 (0.75, 0.9) |
| CK-MB | 0.79 (0.71, 0.86) | 0.84 (0.76, 0.91) |
| eGFR | 0.81 (0.73, 0.88) | 0.83 (0.75, 0.9) |
| GDF-15 | 0.8 (0.72, 0.88) | 0.82 (0.75, 0.9) |
| BNP | 0.78 (0.71, 0.86) | 0.83 (0.75, 0.9) |
