# Supplementary material for: GDF-15 predicts cardiovascular events in acute chest pain patients
Source: PLoS One. 2017 Aug 3;12(8):e0182314. doi: 10.1371/journal.pone.0182314 (PMC5542604; doi:10.1371/journal.pone.0182314)
Supplement: S5 Table — NRI denotes net reclassification index. eGFR denotes estimated glomerular filtration rate, BNP denotes B-type natriuretic peptide, GDF denotes growth differentiation factor. (DOC) [file pone.0182314.s006.doc]

### **S5 Table.**

|  | **cont. NRI** | **cont. NRI: p-value** | **IDI** | **IDI: p-value** | **rel. IDI** |
| --- | --- | --- | --- | --- | --- |
| Troponin I | 0.526 | p < 0.001 | 0.0343 | p = 0.054 | 0.1275 |
| Creatinin kinase | 0.264 | p = 0.027 | 0.0123 | p = 0.11 | 0.0497 |
| Creatinin kinase-MB | 0.538 | p < 0.001 | 0.0267 | p = 0.028 | 0.1093 |
| eGFR | 0.085 | p = 0.254 | -0.0025 | p > 0.5 | -0.0094 |
| GDF-15 | 0.352 | p = 0.004 | 0.0354 | p = 0.006 | 0.1456 |
| BNP | 0.415 | p < 0.001 | 0.0202 | p = 0.028 | 0.0769 |
