# Supplementary material for: GDF-15 predicts cardiovascular events in acute chest pain patients
Source: PLoS One. 2017 Aug 3;12(8):e0182314. doi: 10.1371/journal.pone.0182314 (PMC5542604; doi:10.1371/journal.pone.0182314)
Supplement: S6 Table — NRI denotes net reclassification index. Event was defined as Death or myocardial infarction in the six months following baseline presentation. (DOC) [file pone.0182314.s007.doc]

### **S6 Table.**

|  | **Reclassified upwards** | **Reclassified downwards** | **NRI** |
| --- | --- | --- | --- |
| **GRACE Score + TnI** |  |  |  |
| Kaplan-Meier rate of events | 0.32 | 0.011 |  |
| Expected number of events | 164.5 | 5.7 | 6.8 % |
| Expected number of non-events | 349.5 | 508.3 | 3.1 % |
| NRI + p-value |  |  | **9.9 %**; p: 0.09229 |
| CI: NRI |  |  | ( -1.6 %, 21.4 %) |
| **GRACE Score + CK** |  |  |  |
| Kaplan-Meier rate of events | 1 | 0.018 |  |
| Expected number of events | 209 | 3.8 | -9.1 % |
| Expected number of non-events | 0 | 205.2 | 6.4 % |
| NRI + p-value |  |  | **-2.7 %**; p: 0.49219 |
| CI: NRI |  |  | ( -10.4 %, 5 %) |
| **GRACE Score + CKMB** |  |  |  |
| Kaplan-Meier rate of events | 0.54 | 0.013 |  |
| Expected number of events | 285.1 | 6.9 | 3.6 % |
| Expected number of non-events | 242.9 | 521.1 | 3.6 % |
| NRI + p-value |  |  | **7.2 %**; p: 0.00539 |
| CI: NRI |  |  | (2.1 %, 12.3 %) |
| **GRACE Score + eGFR** |  |  |  |
| Kaplan-Meier rate of events | 0.159 | 0.088 |  |
| Expected number of events | 35.8 | 19.8 | -6.6 % |
| Expected number of non-events | 189.2 | 205.2 | 5.8 % |
| NRI + p-value |  |  | **-0.8 %;** p: 0.81065 |
| CI: NRI |  |  | ( -7.1 %, 5.5 %) |
| **GRACE Score + GDF-15** |  |  |  |
| Kaplan-Meier rate of events | 0.127 | 0.094 |  |
| Expected number of events | 41.5 | 30.7 | 10.9 % |
| Expected number of non-events | 285.5 | 296.3 | 1.6 % |
| NRI + p-value |  |  | **12.5 %**; p: 0.04484 |
| CI: NRI |  |  | (0.3 %, 24.6 %) |
| **GRACE Score + BNP** |  |  |  |
| Kaplan-Meier rate of events | 0.382 | 0.011 |  |
| Expected number of events | 265.5 | 7.6 | 6.7 % |
| Expected number of non-events | 429.5 | 687.4 | 3.3 % |
| NRI + p-value |  |  | **10 %**; p: 0.00246 |
| CI: NRI |  |  | (3.5 %, 16.4 %) |
